# From prototype to national standard: a governance framework for scaling simulation-based bronchoscopy training

**DOI:** 10.1186/s41077-026-00452-9

**Published:** 2026-06-08

**Authors:** Michal Pirozynski, Jack Jakimowicz

**Affiliations:** Centre of Postgraduate Medical Education (CMKP), Marymoncka 99/103, 01-813 Warsaw, Poland

**Keywords:** Bronchoscopy, Simulation-based education, Postgraduate medical education, Implementation science, Procedural training, Quality assurance

## Abstract

**Background:**

Simulation-based education can improve procedural training, but national adoption depends on governance, scalability, quality assurance, and alignment with postgraduate certification requirements. This Advancing Simulation Practice report describes how bronchoscopy simulation developed from a pilot educational initiative into a mandatory national component of postgraduate pulmonary medicine training.

**Main body:**

We present a practice-based implementation account of a national reform in procedural training, using bronchoscopy as the index procedure. Rather than testing an effectiveness hypothesis, we examine programme design, governance, scale-up, instructor development, quality assurance, and regulatory embedding. The Consolidated Framework for Implementation Research and the RE-AIM framework were used to structure reflection on implementation determinants and programme outcomes, rather than as prospectively applied measurement instruments. The national simulation programme trained 402 physicians across procedural specialties, supported by 85 nationally certified instructors, and delivered 184 structured training cycles comprising 5,999 instructional hours. The bronchoscopy component enrolled 186 pulmonary specialty trainees, of whom 185 completed the course; 46 instructors were certified to deliver bronchoscopy training. Post-course evaluations showed high perceived relevance and increased self-reported confidence, which are interpreted as acceptability and early implementation indicators rather than evidence of clinical competence. Following national evaluation, bronchoscopy simulation was incorporated in 2024 as a mandatory component of postgraduate specialisation training in pulmonary diseases. Scale-up was supported by national-level European Union structural funding for health workforce development and simulation infrastructure, including POWER project POWR.04.03.00-00-0291/16, in which bronchoscopy formed one component of broader endoscopic simulation capacity.

**Conclusion:**

The main contribution of this programme was the governance pathway that enabled simulation to progress from local innovation to national training policy. Sustainable implementation depended on staged scale-up, explicit curriculum design, instructor certification, common standard operating procedures, external quality assurance, distributed simulation-centre capacity, and alignment with regulatory postgraduate training requirements. Future evaluation should examine clinical transfer, workplace performance, and patient-safety outcomes.

## Background

Simulation-based education has consistently been shown to improve procedural performance, particularly when learning is structured around feedback, repetition, progressive task difficulty, and opportunities for deliberate practice [1–5]. More recent bronchoscopy literature confirms that simulation-based training improves simulated bronchoscopy performance, while also emphasising that evidence of transfer to patient care remains more limited and requires further study [5, 6].

The national bronchoscopy simulation programme described in this manuscript was developed as part of a broader national reform integrating simulation-based procedural training into postgraduate pulmonary medicine education in Poland. The educational design of the programme drew on mastery learning, deliberate practice, Kolb’s experiential learning theory, and structured feedback.

Despite this strong educational foundation, large-scale integration of simulation into regulated postgraduate training systems remains challenging. Several national and multicentre bronchoscopy simulation initiatives have now been reported, including the Dutch nationwide bronchoscopy simulation programme [6]. However, detailed descriptions of the governance mechanisms, funding structures, instructor certification systems, quality assurance processes, and policy pathways that enable national implementation remain relatively uncommon.

**Fig. 1:**
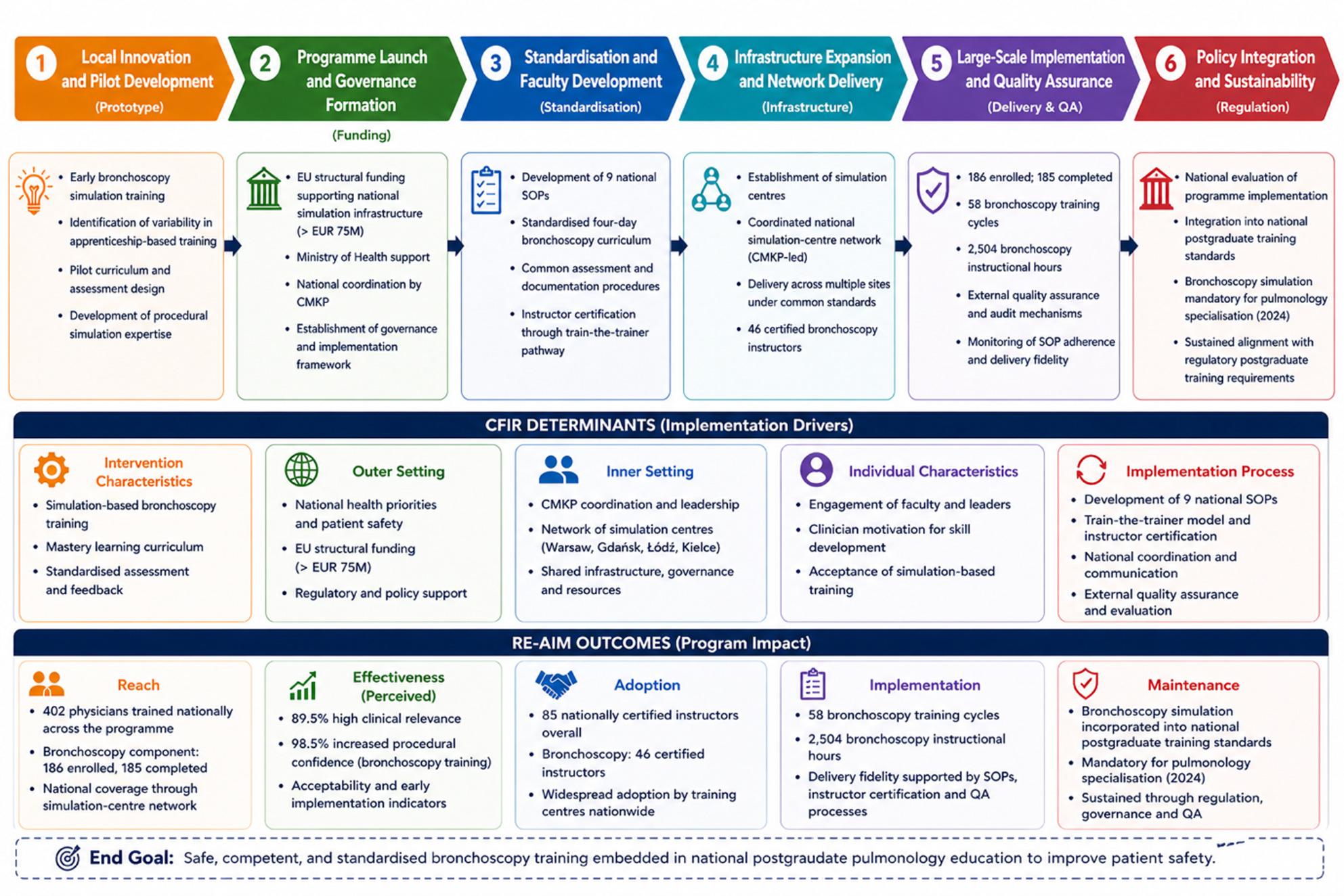
Implementation pathway, determinants, and outcomes of national bronchoscopy simulation training This figure presents the overall implementation pathway from local pilot development to national policy integration. The upper panel illustrates the staged scale-up process. The middle panel maps key implementation determinants using the Consolidated Framework for Implementation Research (CFIR; intervention characteristics, outer setting, inner setting, individual characteristics, and implementation process). The lower panel summarises programme outcomes using the RE-AIM framework (Reach, Effectiveness, Adoption, Implementation, and Maintenance). Together, the figure demonstrates how standardisation, governance, instructor certification, and quality assurance enabled national adoption and sustainability

The Consolidated Framework for Implementation Research (CFIR) identifies multilevel determinants of implementation success, including intervention characteristics, outer setting, inner setting, individual roles, and implementation process [7]. The RE-AIM framework - Reach, Effectiveness, Adoption, Implementation, and Maintenance - provides a complementary way to interpret whether an intervention is taken up, delivered with fidelity, and sustained at population or system level [8]. In this manuscript, these frameworks were used retrospectively to structure implementation reflection rather than prospectively to define measurement instrument (Fig. 1).

International patient safety policies further emphasize the need for structured competency validation in high-risk clinical procedures [9]. Embedding simulation within governance and certification frameworks therefore represents not only an educational strategy but also a system-level patient safety intervention.

The national rationale for reform also reflected a system-level gap in procedural skills certification. Before this programme, postgraduate procedural training in Poland did not include a unified national certification pathway for the acquisition and maintenance of selected endoscopic skills. As a result, trainees’ exposure depended heavily on local case volume, supervisor availability, and institutional resources, creating a need for a standardised simulation-based pathway that could support comparable preparation across training centres.

This manuscript is presented as an implementation-focused practice report rather than a hypothesis-driven research study. It describes the implementation and national scale-up of bronchoscopy simulation training integrated into postgraduate pulmonary medicine education in Poland. It focuses on how the programme was designed, governed, expanded, quality assured, and embedded into national training requirements. The intended readers are simulation educators, specialty-training leaders, and policy stakeholders seeking to move simulation-based procedural training from local course delivery to system-level adoption.

## Rationale for selecting bronchoscopy

Bronchoscopy was selected as the initial focus of reform because it combines procedural risk, technical complexity, variability in training exposure, and suitability for objective performance assessment.

### Target learners and pre-intervention context

The target learners were physicians in postgraduate specialty training in pulmonary diseases. Before national implementation, bronchoscopy training depended largely on local apprenticeship models, procedural volume, supervisor availability, and variable access to simulation. Although some centres had simulation resources, there was no nationally standardised bronchoscopy curriculum, common instructor certification pathway, unified objective assessment process, or requirement that pulmonary trainees complete simulation before progressing in clinical bronchoscopy training. This variability had educational and ethical implications: opportunity-based patient learning alone could not ensure comparable case mix, supervision, feedback, or procedural complexity before patient contact. Simulation was therefore introduced as a patient-safety mechanism to provide equitable baseline exposure, structured practice, feedback, and assessment before clinical bronchoscopy.

Flexible bronchoscopy is an invasive airway procedure associated with complications such as hypoxemia, bleeding, bronchospasm, pneumothorax, and sedation-related instability [10, 11]. Although complication rates remain acceptable when the procedure is performed by experienced clinicians, professional guidelines emphasize the importance of structured training and supervised practice to mitigate procedural risk [10, 12]. Variability in complication reporting and quality assurance practices further highlights the need for standardised competence validation.

The procedure requires coordinated navigation within the three-dimensional bronchial tree, real-time image interpretation, continuous physiological monitoring, and ongoing clinical decision-making. These integrated cognitive and psychomotor demands contribute to a steep learning curve for novice trainees.

From a mastery learning perspective, bronchoscopy provides clearly measurable performance endpoints - such as systematic airway inspection and procedural completeness - making it well suited for structured simulation-based training and objective assessment [2, 13].

Training exposure varies across institutions due to differences in case volume, local organisation of respiratory services, and availability of experienced supervisors [5, 10, 12]. Traditional apprenticeship models based primarily on time-dependent clinical exposure or procedure counts cannot ensure uniform competence acquisition at the national level. Simulation therefore offers a means of providing standardised training exposure independent of local procedural volume, while allowing learners to practise airway navigation, equipment handling, anatomical recognition, complication awareness, and procedural decision-making in a controlled environment before performing procedures on patients [5, 6, 12, 14].

## Practice context and implementation approach

We conducted a descriptive, practice-based implementation analysis of a national educational reform that integrated simulation-based bronchoscopy training into postgraduate pulmonary medicine education. This work is best understood as a retrospective implementation case analysis drawing on programme documentation, training records, participant evaluations, instructor reports, and external audit findings, rather than as a prospective effectiveness study. The article is presented as an Advancing Simulation Practice report because its primary contribution is the transferable implementation pathway rather than a controlled comparison of educational effectiveness. Accordingly, the level of inference is descriptive and explanatory, focusing on how implementation was achieved rather than testing causal relationships between intervention and outcomes. No inferential statistical analysis was performed, because the purpose was descriptive implementation reporting.

The guiding practice questions were: [1] how can bronchoscopy simulation be scaled from a pilot initiative to a national training standard; [2] what governance mechanisms are required to maintain fidelity across multiple simulation centres; and [3] what lessons can inform other procedural simulation programmes seeking regulatory integration?

Participants were physicians enrolled in postgraduate specialty training pathways relevant to the national simulation programme. The bronchoscopy component was directed primarily at physicians specializing in pulmonary diseases. Recruitment occurred through the national postgraduate medical education and project infrastructure rather than through a single local training centre. All participants provided written informed consent, including consent for the processing of personal data. The study was approved by the Bioethics Committee of the Medical Centre for Postgraduate Education (No. 102/PB/2019, 13 November 2019).

Participant feedback was collected immediately after course completion using standardised post-course evaluation questionnaires based on a five-point Likert scale. These measures were selected as pragmatic implementation indicators of acceptability, perceived clinical relevance, perceived confidence, and overall training quality during the scale-up phase. They were not intended to measure clinical competence, retention, transfer to workplace performance, or patient outcomes.

## Intervention and curriculum design

The reform introduced a structured bronchoscopy simulation curriculum grounded in mastery learning, deliberate practice, experiential learning, and simulation-design principles [2, 13, 15, 16].

Curriculum content was developed by a multidisciplinary expert group including pulmonologists, bronchoscopy educators, simulation faculty, postgraduate training representatives, and project coordinators. Design decisions were informed by national postgraduate training requirements, local needs assessment, patient-safety priorities, and international bronchoscopy guidance, particularly British Thoracic Society bronchoscopy guidance. Relevant international bronchoscopy education recommendations, including WABIP Bronchoscopy Education Project materials, were also considered where applicable [10, 17]. Expert discussions focused on harmonising patient-safety priorities, postgraduate training requirements, and international bronchoscopy guidance. This process incorporated iterative multidisciplinary expert consultation elements consistent with a modified Delphi-informed consensus approach aimed at harmonising educational objectives, procedural priorities, and implementation standards.

The four-day, 32-hour curriculum included orientation to equipment and simulator use, airway anatomy, bronchoscope handling, systematic airway inspection, recognition of common abnormalities, patient monitoring, complication awareness, procedural decision-making, supervised simulation practice, feedback and debriefing, and objective structured assessment. Trainees progressed through the programme by demonstrating predefined competence benchmarks assessed with objective structured assessment tools. Standardised feedback protocols were incorporated into each training cycle to support consistent formative evaluation and progressive skills development.

Although these tools supported formative progression and standardisation across centres, formal psychometric validation and formal implementation of dedicated bronchoscopy assessment instruments such as BSTAT were beyond the scope of this implementation-focused report.

### User needs and design requirements

The principal user need was to give pulmonary trainees reliable access to structured bronchoscopy practice before patient contact, regardless of local clinical volume or institutional resources. These needs were identified through review of existing postgraduate training requirements, observed variability in local bronchoscopy training capacity, consultation with pulmonary and simulation educators, and project-level assessment of simulation-centre readiness. The design requirements were therefore: national accessibility, standardised curriculum content, reproducible assessment, certified instructors, compatibility with existing postgraduate training regulations, and feasibility of delivery across a distributed network of simulation centres.

## Governance architecture

**Fig. 2:**
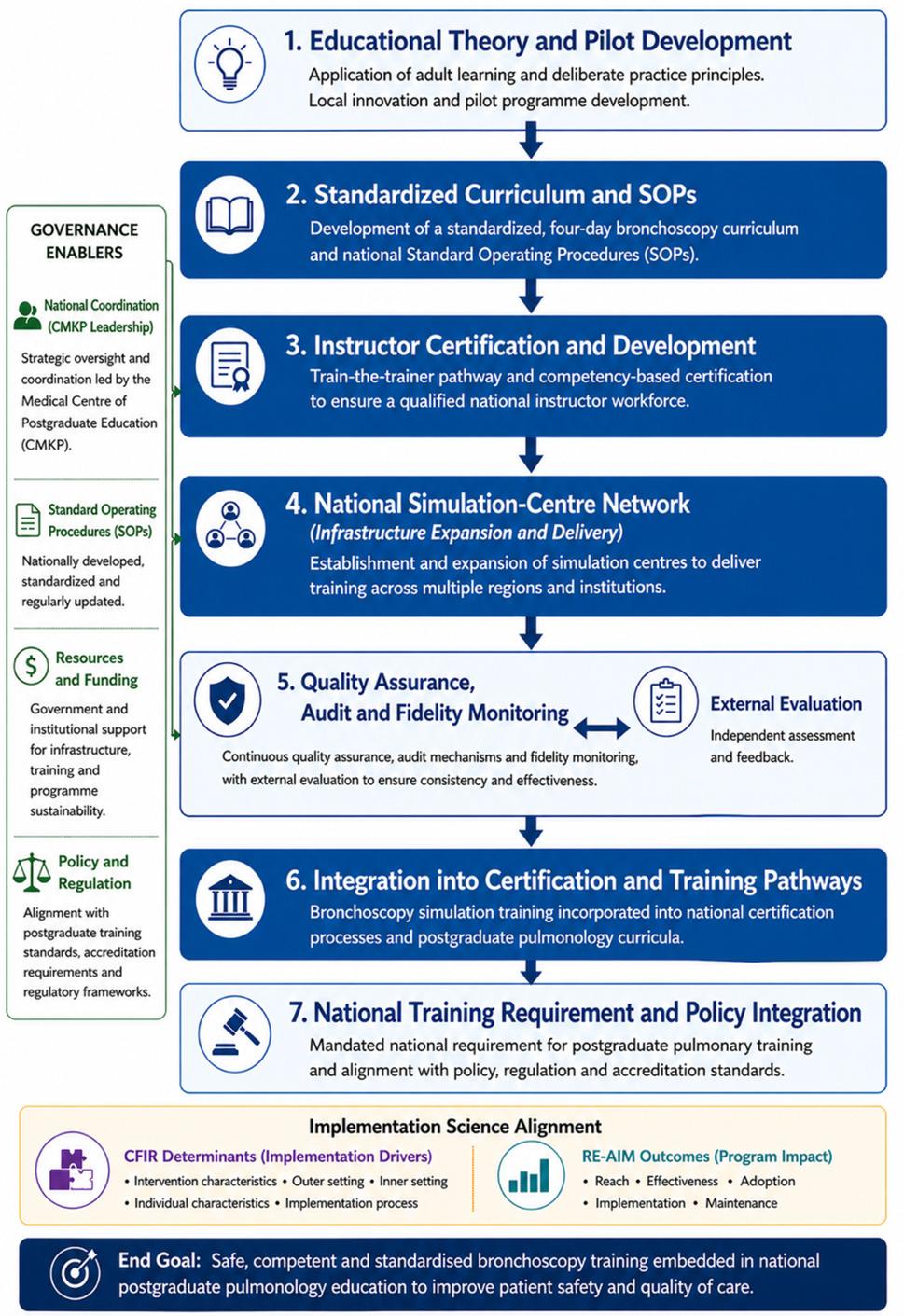
Governance pathway for national scale-up of bronchoscopy simulation training This figure illustrates the governance architecture underpinning national scale-up. It shows how educational design was translated into operational standards through curriculum development, national Standard Operating Procedures (SOPs), and instructor certification. These elements supported delivery across a coordinated network of simulation centres, reinforced by quality assurance, audit, and external evaluation. The central insight is that governance mechanisms enabled consistent, reproducible delivery and facilitated integration into postgraduate training requirements

National scale-up required formal standardisation before expansion. The governance architecture underpinning national scale-up is summarised in Fig. 2. Nine national Standard Operating Procedures (SOPs) were developed to define curriculum structure, procedural techniques, instructional methodology, assessment criteria, instructor qualification standards, equipment requirements, documentation processes, and quality assurance mechanisms. The SOPs served as the operational bridge between educational design and reproducible national delivery.

The SOP-development process was informed by literature review, analysis of existing procedures in reference simulation centres, experience from international simulation-centre networks, and study visits to external partner institutions. An interdisciplinary expert group coordinated by CMKP translated these inputs into operational standards covering organisation of training, network management, recruitment, instructor preparation, audio-video systems, learning-platform management, bronchoscopy delivery, and other endoscopic simulation domains.

A coordinated Network of Simulation Centres was established to ensure national accessibility while maintaining fidelity to standardised educational and procedural protocols.

The network model combined distributed delivery with central coordination. Regional centres were developed with simulation laboratories, debriefing rooms, advanced endoscopic simulation equipment, and audio-video systems that enabled remote supervision and ongoing methodological support from CMKP faculty. This infrastructure allowed local access while preserving a common national standard for curriculum delivery and assessment.

Instructor certification was formalized through a national train-the-trainer pathway designed to ensure instructional quality and consistency of assessment. Faculty development preceded infrastructure expansion so that training standards, feedback language, assessment expectations, and documentation procedures were stabilized before delivery was distributed across multiple centres.

External audit mechanisms were implemented to reinforce compliance, transparency, and adherence to nationally defined procedures.

During the testing and refinement phase, external evaluation reviewed the developed standards, training documentation, trainee evaluation reports, and supervisory reports from CMKP instructors overseeing regional-centre delivery. This audit process supported the conclusion that the standards were suitable for broader implementation and that a certification pathway based on common simulation standards was feasible.

Operationally, governance responsibilities were distributed between central and regional levels. The central coordinating body (CMKP) was responsible for curriculum definition, SOP development and revision, instructor certification standards, and oversight of quality assurance processes. Regional simulation centres were responsible for programme delivery in accordance with SOPs, local logistics, and documentation of training activities. Compliance was monitored through periodic external audits, review of training documentation, and supervisory reporting. SOP updates were managed centrally and disseminated across the network to maintain consistency. This structure enabled distributed delivery while preserving national standardisation.

Instructor certification required completion of a structured train-the-trainer programme including calibration in assessment standards, feedback delivery, and adherence to SOP-defined instructional methods. Ongoing quality assurance included periodic review of training sessions, documentation audits, and supervisory oversight by central faculty. Where deviations from SOPs were identified, corrective actions included targeted instructor feedback, additional faculty development, and, where necessary, temporary suspension of training delivery until compliance was restored. These mechanisms were designed to maintain consistency of training quality across the national network.

### Stages of scale-up from pilot to national implementation

**Fig. 3:**
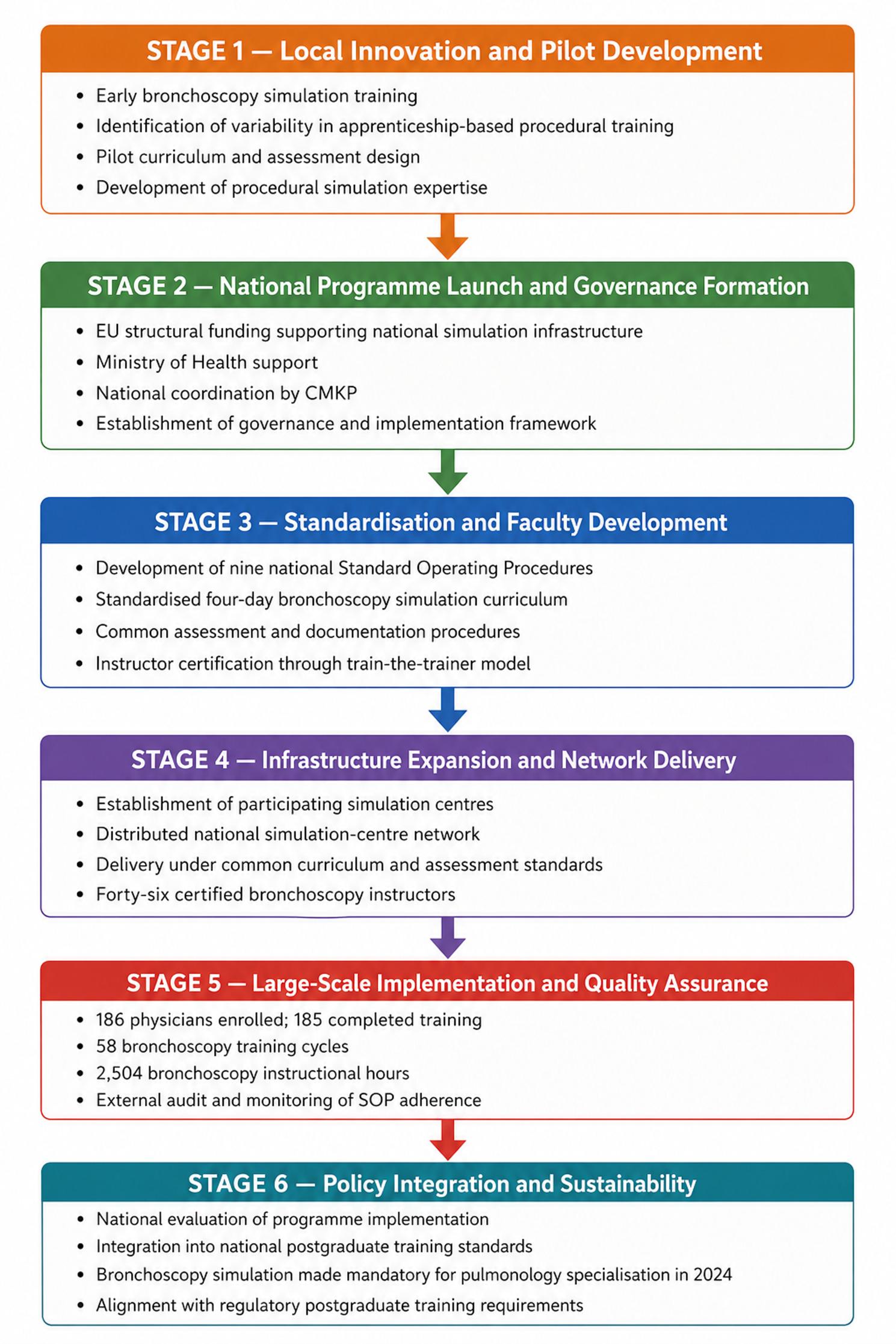
Staged implementation pathway for national bronchoscopy simulation training This figure provides a simplified operational overview of the staged scale-up process, complementing Fig. 1, which integrates implementation theory (CFIR and RE-AIM) with programme outcomes. It illustrates progression from pilot development through standardisation, faculty preparation, infrastructure expansion, and national implementation to policy integration. A key sequencing insight is that standardisation and instructor development preceded infrastructure expansion, supporting implementation fidelity during national scale-up

The scale-up process occurred in staged phases (Fig. 3). First, the programme team defined the educational problem: variable bronchoscopy exposure and the absence of a national simulation-based competence pathway. Second, a pilot curriculum and assessment model were developed to test feasibility, equipment requirements, faculty roles, documentation, and learner flow. Third, national SOPs were created before broader dissemination, so that sites implemented the same curriculum, assessment expectations, safety procedures, and reporting requirements. Fourth, instructor preparation was prioritized through a train-the-trainer model, because reliable national delivery required a shared faculty standard before infrastructure expansion and growth in learner numbers. Fifth, training was distributed across the simulation-centre network, supported by infrastructure expansion, external audit, and project-level monitoring. Finally, following national evaluation, the bronchoscopy component was incorporated in 2024 into mandatory postgraduate training in pulmonary diseases. This sequence illustrates the central implementation lesson: standardisation and faculty calibration preceded scale-up.

This staged pathway is the main practice contribution of the manuscript: simulation technology enabled skills practice, but governance enabled national adoption.

Operationally, this staged process followed a development-testing-analysis-refinement-recommendation sequence. Curricula and SOPs were first developed centrally, then tested in regional centres after train-the-trainer preparation. Implementation data, participant evaluations, instructor supervisory reports, and external audit findings were then analysed to refine the standards before recommendations were made for wider national implementation. 

## Implementation outcomes

The principal implementation outcomes of the national simulation programme and the bronchoscopy training component are summarised in Table 1. 

**Table 1:** Key implementation outcomes of the national simulation programme and the bronchoscopy training component This table summarises programme scale, training capacity, and early implementation indicators for both the overall national simulation programme and the bronchoscopy-specific component. The national programme included multiple procedural specialties, with bronchoscopy presented here as the index example for this implementation report. Reported participant ratings reflect immediate post-course measures of acceptability, perceived relevance, and self-reported confidence. These indicators should be interpreted as measures of early implementation response rather than objective evidence of clinical competence, skill retention, workplace transfer, or patient outcomes

Indicator	National simulation programme	Bronchoscopy training
Programme scale		
Physicians trained	402	186 enrolled (185 completed)
Certified instructors	85	46
Training cycles	184	58
Instructional hours	5,999	2,504
Acceptability and early implementation indicators		
Participants rating training as highly relevant to clinical practice	-	89.5%
Participants reporting increased self-reported procedural confidence	-	98.5%
Policy impact		
National policy outcome	Simulation integrated into postgraduate training	Mandatory in pulmonology specialisation training (2024)

### Programme reach and training capacity

The national simulation programme trained 402 physicians across procedural specialties, supported by 85 nationally certified instructors. Across the programme, 184 structured training cycles were conducted, representing 5,999 instructional hours delivered within the national simulation network. These figures refer to the broader national programme, not only to bronchoscopy.

For the bronchoscopy component, participating physicians were enrolled in a standardised four-day course with a total duration of 32 instructional hours. This structure ensured uniform exposure to the simulation curriculum across training centres and reduced dependence on local case volume.

Training was delivered through a coordinated national network of simulation centres operating under common curricula, instructor certification, and quality assurance procedures. All training cycles were conducted in accordance with the national Standard Operating Procedures defining curriculum structure, instructional methodology, and assessment processes.

### Bronchoscopy-specific implementation

Bronchoscopy training was implemented within the broader national simulation programme as a specialty-specific component for physicians in pulmonary specialty training. In total, 186 physicians enrolled in bronchoscopy simulation training, and 185 completed the programme after one participant withdrew. Post-course evaluation demonstrated high acceptability and perceived educational value: 89.5% of participants rated the training as highly relevant to clinical practice, and 98.5% reported increased procedural confidence. These findings are presented as immediate learner-reaction and implementation-acceptability data, not as direct evidence of competence. In addition, 46 instructors were certified for the bronchoscopy training component, supporting delivery across a multicentre simulation network.

### Participant evaluation

Immediate post-course evaluations supported high participant acceptance of the training programme. These findings should be interpreted as indicators of learner reaction, perceived relevance, and self-efficacy rather than evidence of sustained competence, workplace transfer, or patient benefit.

### System-level outcome

Following national evaluation of the programme, bronchoscopy simulation training was incorporated in 2024 as a mandatory component of postgraduate specialisation training in pulmonary diseases. The programme was implemented across participating training centres with consistent adherence to the standardised curriculum and assessment procedures. EU structural funding of more than EUR 75 million, through European Union cohesion-policy instruments supporting health workforce development and educational infrastructure, supported the broader national simulation-centre infrastructure and training capacity for endoscopic procedures; bronchoscopy was one component of this wider national investment rather than the sole recipient of the funding.

## Discussion

### Principal practice lesson

This article describes how bronchoscopy simulation was moved from an educational innovation to a nationally embedded training requirement. The principal practice lesson is not that learners reported high satisfaction or confidence, but that sustainable national implementation depended on a governance architecture capable of standardising delivery, assessment, and quality assurance across centres.

### From course design to system design

Many simulation initiatives begin as locally successful courses but struggle to scale because course quality depends on individual champions, local resources, or informal faculty knowledge. In this programme, scale-up was possible because educational design was translated into operational standards. The nine SOPs, instructor certification pathway, common documentation, and external audit mechanisms made it possible to deliver the same educational model across a geographically distributed network. For readers seeking to adapt this model, the transferable sequence is to define the patient-safety problem, standardise curriculum and assessment, calibrate instructors, establish documentation and audit processes, and only then expand delivery across sites.

### Educational theory and curriculum implications

Mastery learning and deliberate practice were important because bronchoscopy requires repeated performance, feedback, and progression toward defined standards. However, the programme was not limited to psychomotor skill acquisition. The four-day curriculum also used experiential learning, feedback, debriefing, and progressive integration of clinical reasoning. This broader design was necessary because safe bronchoscopy requires not only scope navigation but also anatomical recognition, patient monitoring, complication awareness, and procedural judgement. This design is also consistent with Kolb’s experiential learning model, in which learners cycle between concrete experience, reflective observation, abstract conceptualization, and active experimentation within simulation-based procedural training.

### Implementation science interpretation

Viewed through CFIR, implementation was supported primarily by the outer setting (a national policy need for comparable procedural preparation), intervention characteristics (standardised curriculum and SOPs), inner setting (distributed simulation-centre capacity), characteristics of individuals (certified instructors), and implementation process (sequenced development, testing, refinement, and scale-up). Viewed through RE-AIM, the programme achieved reach through national training-centre access, adoption through instructor and centre participation, implementation through SOP-based delivery, and maintenance through incorporation into mandatory postgraduate pulmonology training.

### Relation to other bronchoscopy simulation programmes

Other national and multicentre bronchoscopy simulation programmes have been reported, including recent Dutch work demonstrating improvement in simulated bronchoscopy competence after nationwide training [6]. The present contribution differs in emphasis: it focuses less on demonstrating short-course effectiveness and more on the governance conditions that allowed a simulation curriculum to become a national training requirement.

### Equity, access, and patient safety

The distributed national network was also intended as an equity intervention. By creating common curricula, instructor standards, and simulation-centre capacity, the programme aimed to reduce geographic and institutional differences in access to structured procedural training. Although formal evaluation of equity outcomes, such as regional access, trainee demographics, or differential participation, was not conducted, the national distribution of centres was designed to improve accessibility. This is directly relevant to patient safety because trainees should not depend solely on local case volume or informal apprenticeship opportunities before undertaking invasive airway procedures. Future programme monitoring should include explicit evaluation of equity, diversity, and inclusion outcomes.

### Limitations

Several limitations should be acknowledged. First, this is a practice-based implementation account rather than a controlled effectiveness study. Second, participant feedback was collected immediately after course completion and therefore captures acceptability, perceived relevance, and self-efficacy rather than competence, retention, workplace transfer, or patient outcomes. Third, the implementation occurred within a specific Polish postgraduate regulatory and funding context, which may limit direct transferability. Fourth, although SOPs and external audit supported fidelity, this report does not present independent psychometric validation of all assessment tools or longitudinal data linking simulation performance to clinical bronchoscopy outcomes. Fifth, because the evaluation was designed for implementation monitoring rather than comparative effectiveness testing, no inferential statistical analysis was undertaken.

### Future evaluation

Future research should examine whether national simulation requirements translate into improved workplace performance and patient-safety outcomes. Potential approaches include validated bronchoscopy assessment tools, procedure-log analysis, supervisor evaluations, workplace-based assessment, video review, simulator-retention testing, complication monitoring, and longitudinal follow-up after trainees enter supervised clinical practice.

## Conclusion

In conclusion, national integration of bronchoscopy simulation depended less on simulation technology alone than on governance. The transition from prototype to national standard required staged scale-up, instructor certification, common SOPs, external quality assurance, distributed infrastructure, and regulatory alignment. For other simulation programmes seeking durable system-level impact, the transferable lesson is to design simultaneously for learning, implementation fidelity, equity of access, and regulatory sustainability.

## Data Availability

No datasets were generated or analysed during the current study.
